# Predicting protein cascade expression from H&E images

**DOI:** 10.1371/journal.pcbi.1014262

**Published:** 2026-05-04

**Authors:** Alejandro Leyva, Abdul Rehman Akbar, Muhammad Khalid Khan Niazi

**Affiliations:** Department of Pathology, The Ohio State University, Columbus, Ohio, United States of America; Leibniz University Hanover, GERMANY

## Abstract

Protein expression within oncogenic or suppressive pathways is a hallmark indicator of oncogenesis. While traditional AI models in digital pathology attempt to predict singular proteins, there is a need to predict the downstream expression of proteins to indicate the propagation of signals. RNA expression provides novel information, but does not provide information about the downstream propagation of protein signals or whether those signals are functional. Using Reverse Phase Protein Array (RPPA) data with whole-slide images (WSIs) from the publicly available Cancer Genome Atlas Breast Adenocarcinoma dataset (TCGA-BRCA), we predict the expression of five key proteins identified from the apoptosis cascade, using DNA damage and repair (DDR) cascades as a biological control. Furthermore, we examine the performance of patch- level Vision Transformers (ViT) on the regression task, which was tested against the designed cellular-level ViT, CellRPPA. Our results demonstrate that patch-level vision transformers were unable to obtain statistically significant predictive results, achieving R-squared values < 0.1 for all folds. In addition, CellViT obtained R-squared values >0.1 in all five test folds. We also show that morphologically indicative cascades, such as the apoptosis cascade, provide significantly higher performance compared to the DDR cascade.

## Author summary

We developed a method to estimate how groups of proteins behave inside tumors using standard tissue images that are routinely collected in clinical care. Rather than focusing on single molecules, we asked whether it is possible to capture broader biological processes such as how cells respond to stress or initiate programmed cell death directly from visual patterns in tissue structure. To do this, we trained a computational model to learn relationships between image features and protein measurements from the same tumors.

We found that certain biological processes leave detectable signatures in tissue architecture, allowing the model to partially recover protein activity from images alone. However, this relationship is not complete, and much of the variation remains unexplained, highlighting the complexity of tumor biology.

Our work suggests that widely available pathology images may contain underused in- formation about underlying molecular processes. In the future, approaches like this could help provide additional biological insight in settings where direct molecular measurements are unavailable, supporting more informed research and potentially guiding clinical decision making.

## 1 Introduction

Breast adenocarcinoma is a highly documented disease and can be associated with a variety of genetic mutations that result in the propagation or suppression of protein signals [1]. Pathways can be intrinsically mediated, whereby the stimulus resulting in signal transduction is intracellular, or extrinsic. Within adenocarcinoma, common mutations in proteins such as TP53 result in the suppression of apoptotic signals by mutating the DNA-binding domain, resulting in dysfunctional proteins [2]. Other mutations, such as those in *PIK3CA* or *MYC*, result in the enrichment and propagation of the PIK3CT/AKT pathways by mutating kinase domains, leading to the overexpression of these proteins [3]. Apoptosis is responsible for regulating programmed cell death and is characterized by shrinkage, pyknosis, and reorganization of lipid structure [4]. The pathway is both intrinsically and extrinsically mediated by a combination of enzymes, receptors, and transcription factors [5]. Intrinsically mediated apoptosis occurs in two categorical fashions: negative, which is due to the absence of growth factors around a structure resulting in the triggering of cell death, or positive, which may be due to the presence of antigens, radiation, or hypoxia [6]. These changes result in the opening of inner mitochondrial pores due to lost membrane potential, preventing regular cell metabolism and causing the activation of BH3 proteins that detect metabolic stress [7]. As a result, BH3 proteins deactivate anti-apoptotic proteins such as BCL-2 and begin to activate apoptotic proteins such as BAK/BAX, resulting in the transduction of signals to XIAP (X-linked inhibitor of apoptosis protein), which then disinhibits the caspase family. The released caspases then catabolize the cell, resulting in cellular death [8].

The extrinsic pathway performs a similar function but is dependent on ligand–receptor binding of the TRAIL/FasL proteins, which results in the activation of caspases 8 and 10, which then activate the same caspases up to XIAP [9]. Common forms of extrinsic apoptosis include T-cell–mediated apoptosis and immune response [10].

The DNA damage and repair cascade is responsible for the repair of DNA in response to double-strand breaking or transcriptional errors. It has been demonstrated that lower

expression of the DDR cascade is prognostically predictive within breast adenocarcinoma and has been predictive of chemotherapy sensitivity and response [11]. DDR applications within clinical oncology include testing for mutations in genes responsible for double-strand breaking repair, *BRCA1* and *BRCA2* (Breast Cancer Gene 1/2), and can be secondarily characterized by RNA expression. However, the choice of genes to use for prognostic value is still debated [12]. Moreover, the expression of DDR genes is not physically visible via an electron microscope but can indirectly result in abnormal cellular growth or polyploid cells [13]. Genes that are often used to characterize the DDR cascade include *ATM, CHEK2, H2AFX, RAD51, and TP53*, among others. The ataxia–telangiectasia mutated gene (*ATM*) encodes the response to double-strand breaking and senses DNA double-strand breaks [14]. *RAD51* (radiation-sensitive protein 51) is responsible for the invasion of homologous DNA zones to allow for accurate and timely DNA repair and behaves as an ATPase [15]. *CHEK2* (checkpoint kinase 2) is a protein responsible for producing a kinase that cleaves DNA regions and is a radiation-sensitive protein that prevents the cell from entering mitosis upon DNA damage [16]. *CHEK2* has been shown to be a predictive biomarker for multiple organ cancers across Europe and North America [17]. *TP53* is a transcription factor responsible for encoding the p53 antigen, as well as a protein that activates or inhibits apoptotic genes such as *NOXA* [18]. *TP53* (tumor suppressor antigen 53) typically functions in multiple roles to suppress proliferative pathways and regulate cell cycling. *H2AFX* is responsible for regulating nucleosome formation and produces phosphorylative foci to recruit repair factors for double-strand breaks; the H2A family is also responsible for regulating chromatin accessibility and replication timing [19].

These cascades and their expression are typically predicted using RNA expression in the field of bioinformatics [20]. Within the field of digital pathology, gene expression models and molecular subtyping models have been developed using novel deep learning methods, whereby gene expression can be predicted from whole-slide image features [21]. More recently, multi- modal models integrating protein and genetic data are being developed, complementary to spatial transcriptomics and proteomics [22]. While RNA provides a perspective on cascade expression and prognosis, it does not indicate whether genes are translated and produced, or whether the proteins produced are functional [23]. Traditional models in the field have attempted to predict the expression of singular proteins or antigens that can be readily indi- cated from histology, including *HER2* expression and *EGFR* [24,25]; however, most models do not predict intracellular protein expression due to morphological ambiguity and poor generalization across cancers and external datasets. Multimodal models and comprehensive information on protein expression are required to understand protein functionalization for improved prognostic prediction. Information from proteomics contextualizes cellular behvav- ior and patterns in gene expression in systems biology, as cascades are highly interconnected and cross-talk mechanisms of action [26]. Misalignment between the extent of gene expression and the presence of proteins indicates translational or transcriptional dysfunction or deliberate inhibition.

Protein data are gathered from Reverse Phase Protein Array, which uses fluorescent antibodies that bind to the protein of interest [27]. Luminescence reflects antibody-based detection of target protein levels, while absorbance is used to quantify total protein loading for normalization. The sensitivity and accuracy of RPPA depend on the affinity, specificity, and availability of proteins to bind to the provided antibodies and are conditionally accurate. Typically, multimodal analyses between RNA and RPPA within public datasets are not performed due to the time differential at which each assay is performed, rendering direct comparison between protein and RNA expression invalid. When using AI for expression prediction, information is extracted from region/patch level of whole slide images [28]. In recent years, higher resolution image embeddings have been developed to examine Images at the cellular level, and are untested for protein prediction [29]. Since Protein Cascades are generally visible at the cellular level, there is a need to investigate the ability of AI at both the patch level and the cell level to predict the expression of multiple proteins.

In this study, we predict the expression of multiple intracellular proteins in aggregate as a regression task from WSIs. We present a comprehensive algorithm, CellRPPA, inspired by CellEcoNet [30], to compete with conventional patch-level algorithms. This study offers two novel investigations: i) differences between cell-level and patch-level resolution in deep learning for protein expression prediction, and ii) the capacity for deep learning to predict morphologically ambiguous proteins, or proteins that are loosely indicated by histology [31]. As digital pathology continues to expand, there is an ever-growing need for multimodal models and proteomics to provide a comprehensive perspective on disease progression.

## 2 Materials and methods

From the publicly available Cancer Genome Atlas (TCGA) Breast Adenocarcinoma dataset (TCGA-BRCA), 919 RPPA samples were paired with whole-slide images. RPPA measure- ments were defined at the case level, yielding a 1:1 correspondence between each patient and a single protein cascade score. Multiple WSIs per case were included during training as independent inputs, and slide-level predictions were aggregated to obtain a single case- level estimate. Group-wise cross-validation ensured that all slides from a given patient were assigned to the same fold, preventing leakage. Cascades were defined using proteins. Data splitting was performed at the patient level to prevent leakage between training and test sets, and no samples were skipped or had missing information. The apoptosis cascade was defined as the expression of BCL2, BAX, XIAP, and cleaved caspases 3 and 7. This was done to represent the activation of both intrinsic pathways demonstrated by BCL and BAX, as well as the expression of the encompassing caspases and regulatory inhibitors. The DDR cascade was used as a morphologically ambiguous protein cascade to compare against the performance of apoptosis, which is microscopically noticeable. The proteins chosen to rep- resent the DDR cascade include H2AFX, CHEK2, TP53, TP53 BP1, and ATM; however, this is acknowledged to be a limited representation of the DDR cascade and was chosen to represent hallmark genes associated with prognostic value in breast adenocarcinoma.

The RPPA scores for the relative intensity of each protein were then summed and z-scored across each protein. If any protein that was part of the cascade was not included within a sample, the sample was excluded entirely. Cascade scores were defined as the sum of z- scored protein abundances to provide a compact proxy for coordinated pathway-associated protein expression. This formulation was not intended to model regulatory directionality or mechanistic pathway activity (e.g., opposing pro- and anti-apoptotic effects), but rather to capture aggregate multivariate protein signal as a supervised learning target. We acknowl- edge that this approach does not resolve antagonistic relationships among pathway members. A directionality-aware formulation, such as signed weighting or graph-based aggregation of protein interactions, represents an important future extension to more faithfully model reg- ulatory dynamics. The CellRPPA model is CellEcoNet reengineered with a regression head, and the GitHub repository for the model is included for both CellEcoNet and CellRPPA. Cell embeddings are derived from localized image regions, but CellRPPA is not simply a higher-resolution patch model; it integrates cell-level features within a structured hierarchi- cal aggregation that captures both local and contextual information. The patch-level ViT baseline already tests patch-only input under the same task and performs worse, indicating that the improvement is not explained by resolution alone. Cell embeddings were extracted using Trident and CellViT++, at 20 × magnification, and cell types were not determined.

The apoptosis and DDR tasks were performed separately, and training and evaluation took 150 hours for each task. The patch-level ViT uses a standard ViT-S/16 with cross-fold validation, where the DDR and apoptosis tasks were tested and validated separately and were not backpropagated jointly. We selected a standard ViT-S/16 baseline to isolate the effect of resolution and aggregation strategy rather than architectural optimization. Implementation parameters are shown in Table 1 for the S/16 vision transformer.

**Table 1:** Training configuration and hyper parameters used for regression.

Parameter	Value
Epochs	50
Number of folds	5
Batch size	1
Maximum patches per case	64
Learning rate	1 *×* 10 ^*−* 4^
Weight decay	0.05
Frozen transformer blocks	9
Random seed	42

**Fig 1:**
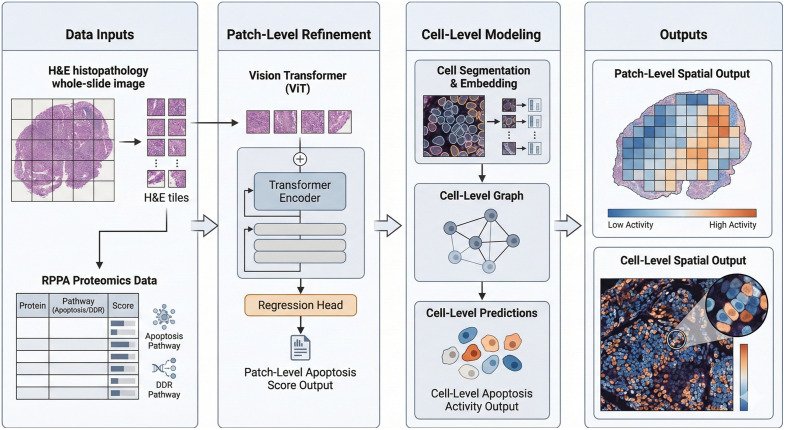
Overview of the cascade prediction framework. Whole-slide H&E images and RPPA-derived protein cascade scores serve as inputs. Patch-level modeling uses a Vision Transformer (ViT) to extract tile embeddings and regress pathway-level protein activity, while cell-level modeling performs cell segmentation, embedding, and graph construction to predict apoptosis activity at cellular resolution. The outputs illustrate spatially resolved pathway activity maps at both patch and cell scales, enabling comparison between coarse tissue-level predictions and fine-grained cellular cascade expression.

All parameterizations for both models are provided within anonymized shell scripts and should be able to run, provided that the necessary label files are available. Fig 1 shows the workflow for the study design, starting from the development of ground truth through the evaluation of each model. All computational experiments were performed on the Ohio Supercomputer on Nvidia A100 GPUs. All analyses were performed on the entire cohort, and the RPPA analytics were derived from the RPPA CSV using matplotlib, NumPy, and SciPy.

## 3 Results

**Fig 2:**
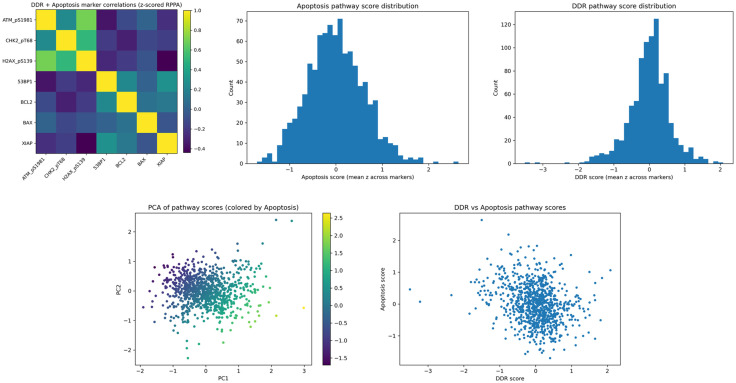
Proteomics and label-level analytics for apoptosis and DDR RPPA-derived path- way scores. Top row: inter-marker correlation structure and pathway score distributions. Bottom row: low-dimensional structure of pathway scores and cross-cascade correlation (control relationship between DDR and apoptosis). **(A)** Marker correlation heatmap (z-scored RPPA), (B) apoptosis score distribution, **(C)** DDR score distribution, **(D)** PCA of pathway scores colored by apoptosis, and **(E)** DDR versus apoptosis pathway scores.

The labels for the protein scores were assigned by case, and each case was assigned into fold-wise training, testing, and validation cohorts for evaluation over 100 epochs per fold. Initial analysis presents results on the expression of proteins defined within each sample as aggregated scores, as shown in Fig 2. Fig 2A shows the correlation heatmap of protein abundance co-variation across each cascade. Proteins included within the apoptosis cascade exhibit moderate coordinated abundance among their counterpart proteins, consistent with shared cascade-level behavior. *BAX* and *XIAP* show very low co-variation, which is biolog- ically plausible given their opposing regulatory roles. Interestingly, *BCL2* and *TP53 BP1* show stronger abundance correlation, exceeding 50% Pearson correlation, while *TP53 BP1* shows negative abundance correlation with other DDR-associated proteins within its defined cascade. *TP53 BP1* also shows a strong correlation with *XIAP* abundance while exhibiting low co-variation with *BAX*. *BCL2* and *BAX* show low abundance correlation, which is consistent with the known biological roles of these proteins.

Within the DDR cascade, *TP53 BP1* shows lower co-variation with DDR-associated pro- teins, including *ATM*, *CHEK2*, and *H2AFX*. The abundance correlation between *ATM* and all other DDR proteins, except *TP53 BP1*, is remarkably strong, exceeding 80% with *H2AFX* and 50% with *CHEK2*. The correlation heatmap shows little overlap between DDR and apoptosis cascade abundance patterns and often demonstrates negative cross-cascade correlations. There is a small positive correlation between *BAX* and *ATM*, while most other cross-cascade correlations are below zero.

Fig 2B shows the standard Gaussian distribution of apoptosis pathway scores, with no observable skew and larger variance from the median. Fig 2C shows the DDR score distribution, which maintains a standard Gaussian distribution with outliers that are three standard deviations away from the mean, attributed to the absence of expression of any classified DDR gene. Fig 2D shows the PCA of pathway scores using UMAP projections for each sample, where samples that are closer together have similar protein expression profiles. A higher frequency of samples cluster toward minimal variance, while outliers, or cases with higher apoptosis cascade scores, are more dispersed and exhibit greater variance in protein expression, or markedly higher expression of proteins that are typically less expressed. To visualize the relationship between DDR scores and apoptosis, Fig 2E shows a plot that classifies samples by their respective scores. In general, samples with higher apoptosis scores tend to have lower DDR scores, and vice versa. However, a large proportion of samples with apoptosis scores near zero also have DDR scores near zero, suggesting an inverse relationship.

The results for the patch-level ViT on the morphologically ambiguous DDR cascade, used as a control, are shown in Table 2. The model failed to obtain statistically significant correlations in three folds using False Discovery Rate (FDR) (p < 0.05) correction on the Spearman correlation for non-linear correlational analysis. Fold 2 completely failed to obtain any Pearson or Spearman correlation as a result of convergence, while the Mean Absolute Error (MAE) remained relatively high. Mean Squared Error (MSE) did not consistently decline across folds, and the MAE remained stagnant across all folds. The patch-level ViT is shown to predict close to no variance in protein expression within the DDR cohort across all five folds. Variability across folds likely reflects cohort heterogeneity and limited sample size. In addition, the Spearman and Pearson values were nearly identical in some folds, indicating similarity in morphological correlation.

**Table 2:** Performance metrics for DDR pathway prediction across folds. Erroneous values or failed convergences are reported as NaN for all tables.

Fold	Pearson *r*	Pearson *p*	Spearman *ρ*	Spearman *p*	MAE	MSE	*R* ^2^	FDR sig
1	0.1479	0.0870	0.1513	0.0799	0.4219	0.3009	0.0135	False
2	NaN	NaN	NaN	NaN	0.4437	0.4088	-0.0011	False
3	0.1243	0.1510	0.1803	0.0364	0.4286	0.3674	-0.0199	False
4	0.2507	0.0032	0.2507	0.0032	0.4106	0.3170	-0.0019	True
5	0.2169	0.0115	0.1418	0.1010	0.4156	0.3497	0.0320	True

The results for the patch-level ViT’s prediction of apoptosis cascade expression are shown in Table 3. The results are noticeably worse for apoptosis, with only one of the five folds showing significant correlations and three of the five folds showing no correlation at all. The MAE also increased relative to the DDR cascade prediction, exceeding 0.5 in some folds, while the typical range of values is between 0 and 1. The MSE did not remain steady and did not consistently improve across folds; Fold 1 demonstrated the best MSE, which then steadily worsened. The model failed to explain any variance in protein expression and, in Fold 3, produced slightly negative values, demonstrating inadequate capability to predict apoptosis cascade expression, despite apoptosis being morphologically visible and the known correlation between protein expression and cascade expression.

**Table 3:** Performance metrics for apoptosis pathway prediction across folds. Undefined correlation values are reported as NaN.

Fold	Pearson *r*	Pearson *p*	Spearman *ρ*	Spearman *p*	MAE	MSE	*R* ^2^	FDR sig
1	0.0392	0.6515	0.0445	0.6082	0.4184	0.2955	0.0003	False
2	NaN	NaN	NaN	NaN	0.5120	0.4067	-0.0116	False
3	NaN	NaN	NaN	NaN	0.4836	0.3702	-0.0330	False
4	0.2236	0.0089	0.2530	0.0030	0.5302	0.4424	0.0029	True
5	NaN	NaN	NaN	NaN	0.4610	0.3313	-0.0018	False

The results for CellRPPA’s prediction of the apoptosis cascade are shown in Table 4, demonstrating improvement over the patch-level ViT, which indicates that higher resolution can be advantageous for the task. For each fold, the epoch with the lowest validation loss was chosen to represent performance across folds. All folds explained at least 10% of the variance in the protein prediction task, while a substantial portion of folds in both the test and validation sets exceeded 20 or 30%. While the MAE remains consistent across cohorts, there is controlled variance across folds. The Pearson and Spearman correlation coefficients across all cohorts were above 40%, with the exception of one fold in the test set. The MSE remained consistent relative to MAE values across cohorts and folds, ranging between 29–35, with the exception of two instances in the test set.

**Table 4:** Cell-level apoptosis prediction performance across five-fold cross-validation. Re-ported metrics include Pearson correlation, Spearman correlation, mean absolute error (MAE), mean squared error (MSE), and coefficient of determination (*R*^2^).

Split	Fold	Pearson	Spearman	MAE	MSE	R^2^
	Fold 1	0.5734	0.5626	0.3811	0.2321	0.3257
	Fold 2	0.6147	0.6097	0.3846	0.3421	0.3773
Train	Fold 3	0.6009	0.5733	0.3808	0.2315	0.3572
	Fold 4	0.6072	0.5859	0.3906	0.2327	0.3926
	Fold 5	0.5969	0.5819	0.3737	0.2332	0.3515
	Fold 1	0.5000	0.4508	0.4771	0.3584	0.2446
	Fold 2	0.4300	0.4274	0.4144	0.2821	0.1708
Validation	Fold 3	0.4564	0.4579	0.4806	0.3551	0.2064
	Fold 4	0.4736	0.4669	0.4419	0.3083	0.1601
	Fold 5	0.5564	0.5754	0.4326	0.2915	0.3068
	Fold 1	0.4982	0.5199	0.4177	0.2800	0.2414
	Fold 2	0.3770	0.3574	0.4645	0.2332	0.1222
Test	Fold 3	0.4629	0.4688	0.4227	0.2855	0.1659
	Fold 4	0.4454	0.4439	0.4469	0.3135	0.1648
	Fold 5	0.5084	0.4755	0.4220	0.2783	0.2484

Table 5 shows the prediction performance for the DDR cascade using CellRPPA, which demonstrated worse values in comparison to apoptosis prediction performance. Relative to the patch-level tasks, CellRPPA showed no improvement over the patch-level ViT in terms of explained variance. Similar to the patch-level ViT, the Spearman and Pearson correlation coefficients fall within the same range, and the PCC values are similar, if not equal, to the Spearman coefficients. The MAE remained within a consistent range, as observed in the other predictive tasks, and was relatively stable across folds but did not exhibit a noticeable decrease.

**Table 5:** Cell-level DNA damage response (DDR) prediction performance across five-fold cross-validation. Metrics include Pearson correlation, Spearman correlation, mean absolute error (MAE), mean squared error (MSE), and coefficient of determination (*R*^2^).

Split	Fold	Pearson	Spearman	MAE	MSE	R^2^
	Fold 1	0.3200	0.2700	0.4000	0.3110	0.1000
	Fold 2	0.3600	0.3600	0.3900	0.2900	0.1300
Train	Fold 3	0.2700	0.2600	0.4100	0.3400	0.0500
	Fold 4	0.3000	0.3000	0.4200	0.3400	0.0900
	Fold 5	0.0900	0.0500	0.4100	0.3300	0.0000
	Fold 1	0.2400	0.2400	0.4500	0.3540	0.0400
	Fold 2	0.1500	0.1000	0.4500	0.3500	-0.0400
Validation	Fold 3	0.0900	0.1100	0.4100	0.2700	-0.0500
	Fold 4	0.2200	0.2200	0.4300	0.3100	-0.1090
	Fold 5	0.1800	0.2400	0.4000	0.3000	0.0000
	Fold 1	0.1700	0.2000	0.4100	0.3200	0.0000
	Fold 2	0.2200	0.2200	0.4100	0.3500	0.0000
Test	Fold 3	0.1300	0.1200	0.4400	0.3500	-0.0500
	Fold 4	0.0000	-0.0200	0.4500	0.3700	-0.2500
	Fold 5	0.1300	0.1400	0.4800	0.4400	-0.0500

The results demonstrate the failure of patch-level ViTs on multi-protein prediction for apoptosis and DDR. In contrast, for cell-level ViTs (CellRPPA), the model succeeds in pre-

dicting apoptosis but fails on DDR, reflecting the histological ambiguity of these proteins. Validation of protein choices for both DDR and apoptosis demonstrates that the selected proteins behaved within the same cascade and function and also exhibited noticeable co- expression. It is also noted that samples with lower DDR expression had higher apoptosis protein expression as measured by RPPA. Across folds, CellRPPA demonstrated improved predictive performance for apoptosis (R2 = 0.189 ± 0.054) compared to the patch-level ViT (R2 = −0.009 ± 0.015). In contrast, both models failed to explain variance in the DDR task (patch-level: R2 = 0.005 ± 0.019; CellRPPA: R2 = −0.070 ± 0.105), consistent with the morphological ambiguity of DDR-associated proteins.

## 4 Discussion

Analysis of breast adenocarcinoma suggests that, in general, there is an inverse relationship between DDR expression and apoptosis expression, which is validated by the correlation heatmap shown in Fig 2A, indicating that a functional basis exists within breast adeno- carcinoma. Previous studies indicate that the apoptosis and DNA damage response cascades are tightly coupled rather than independent. DDR signaling functions upstream to assess genomic damage and, when repair fails, promotes apoptotic commitment. Following irre- versible activation of executioner caspases, key DDR components are cleaved, effectively terminating DNA repair processes [32]. While a few outliers exist in each cohort, the analysis suggests that most proteins exhibit similar levels of expression within the cohort. We acknowledge that this formulation does not account for opposing regulatory roles within pathways. Future work will incorporate directionality-aware weighting or graph-based ag- gregation to better reflect pathway dynamics.

Apoptosis is well known to be visible under compound or fluorescent microscopy, and it is notable that the patch-level ViT fails to detect and predict cascade expression and performs markedly worse than on the DDR cascade. Performance on the DDR cascade in both trials demonstrates that prediction is not suitable at either resolution, likely due to the inherent morphological ambiguity of DDR expression in histology. DDR expression may reflect replicative stress, aneuploidy, and other phenomena that can manifest in multiple visible forms across different cell types. In contrast, apoptosis has been observed to exhibit a set of well-characterized behaviors that are similar across most cell types. It should be noted that patch-level ViT does not provide sufficient resolution to statistically learn the patterns that characterize apoptosis. These results suggest that cascade prediction is better for proteins with histologically visible behaviors and that higher-resolution representations can be advantageous for such cascades. Future work will extend this model to additional pathways (e.g., Reactome) to assess pathway-specific predictability.

Since cellular models are proven to be capable of predicting aggregate protein activity, it makes sense to move toward modeling co-regulation and interactions within protein cas- cades in a spatially resolved way for better explainability and predictive power. With the rise of spatial transcriptomics in digital pathology, interactions between spatial proteomics and transcriptomics can now be modeled at the cellular level using platforms like Xenium and Orion without temporal mismatch between measurements [33,34]. Prior work has explored gene expression prediction using graph-based neural networks, where gene–gene interactions are modeled through distance-aware edges and weighted connectivity [35]. More importantly, transcriptomics has been used to predict proteomics through deep learning, and given the underlying biological coupling, the inverse direction, predicting transcriptomic states from proteomic signals, is also a reasonable extension [36]. Other approaches include contrastive learning across spatial transcriptomic images to capture differences in tumor microenvironments and cellular composition, enabling gene expression inference from contextual variation [37]. Cellular-level models provide the resolution needed to compare microenvironments within the same sample while also capturing transcriptomic and proteomic interactions locally. As a practical objective, cascade-derived features can be used for drug response prediction and survival modeling, as shown by Reitsam et al., allowing AI systems to capture underlying biological dynamics rather than just static measurements [38]. These cascade features can also be extended to tasks like lymph node metastasis prediction or patient risk stratification based on aggregate expression patterns [39]. That said, there is still a gap in integrating full cascade-level information across modalities. While some work has imputed metabolite profiles from RNA, true integration across metabolomics, proteomics, and transcriptomics remains limited [40,41]. The core idea here is that histology can act as the anchor for integrating these signals, enabling cellular-level proteomic cascade prediction directly from tissue phenotype. But realistically, modeling cascades from phenotype back to genotype in digital pathology is still wide open and far from solved.

This study focuses on establishing proof-of-concept within a controlled dataset. External validation across institutions remains an important direction for future work. Given the known domain shift in histopathology, cross-cohort generalization is non-trivial and warrants dedicated study.

## 5 Limitations

While this study uses the term “cascades” to describe multi-protein prediction, each cascade represents a characterized set of hallmark proteins from canonically observed pathways. Full representation of each cascade would require a substantially larger protein cohort, which would introduce additional noise and increase prediction difficulty. We also note that the model choices used for the patch-level ViT are limited and not fully generalizable, and similar limitations apply to CellViTs. Future studies should derive more generalizable conclusions across omics prediction by conducting broader evaluations of currently available models to assess the advantages of cell-level ViTs on omics tasks. In this study, minimal architectures were used to establish baseline performance for multiprotein prediction.Future work will include comparisons to MIL-based and pathology-optimized architectures.

## 6 Conclusion

Using cell-level ViTs and patch-level ViTs, we predict the expression of hallmark proteins within cascades and assess the advantages and disadvantages of each modeling approach. Our results show that cascade prediction is a viable task and can provide insight into molecular, genetic, and cellular functions and responses. Overall, we consider the performance of CellRPPA modest but promising in comparison to other omics-prediction tasks, though improvement in MAE is needed for viability. We demonstrate that patch-level ViTs do not outperform CellRPPA in microscopically visible cascades and that CellRPPA does not provide an advantage over patch-level models for morphologically ambiguous proteins within the DDR cascade. It can be concluded that CellRPPA does not provide an advantage over patch-level ViTs for cascade prediction when targeting morphologically ambiguous proteins.

Future studies should extend this analysis to additional cascades to better understand the integration of deep learning–derived proteomics into clinical informatics. In addition, cross-cohort analyses are needed to demonstrate generalization across cancer types.
